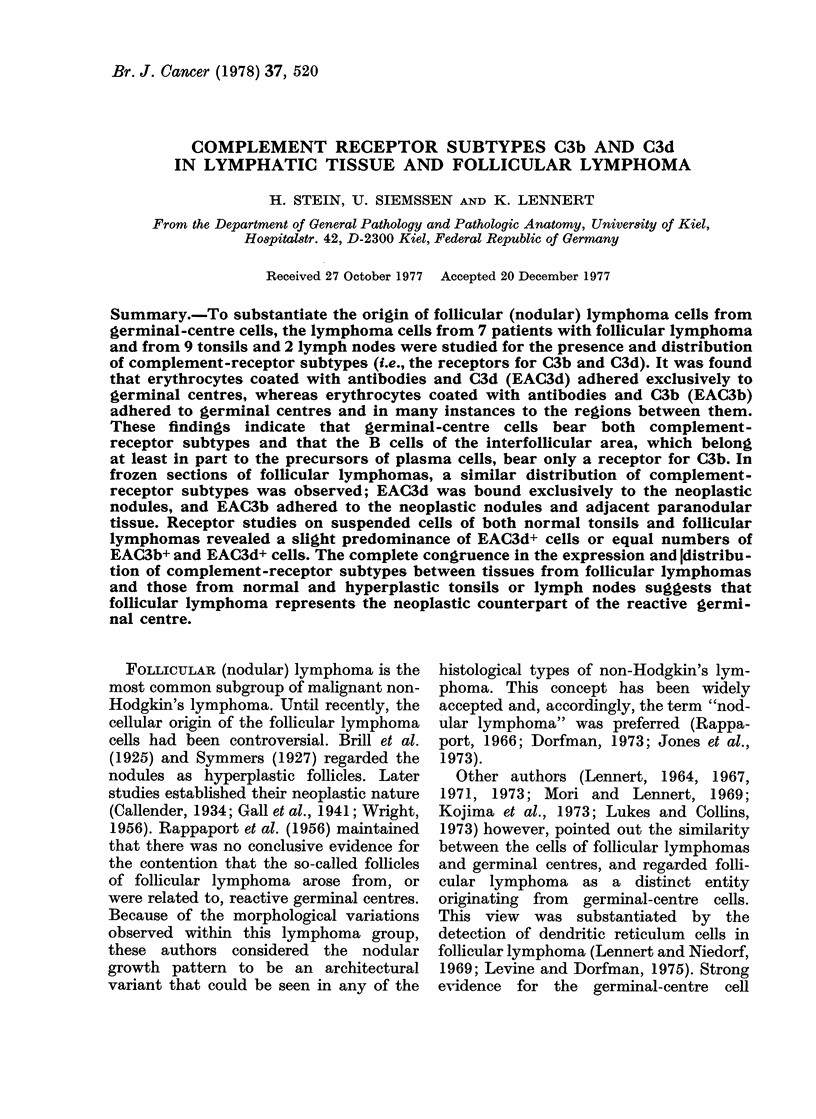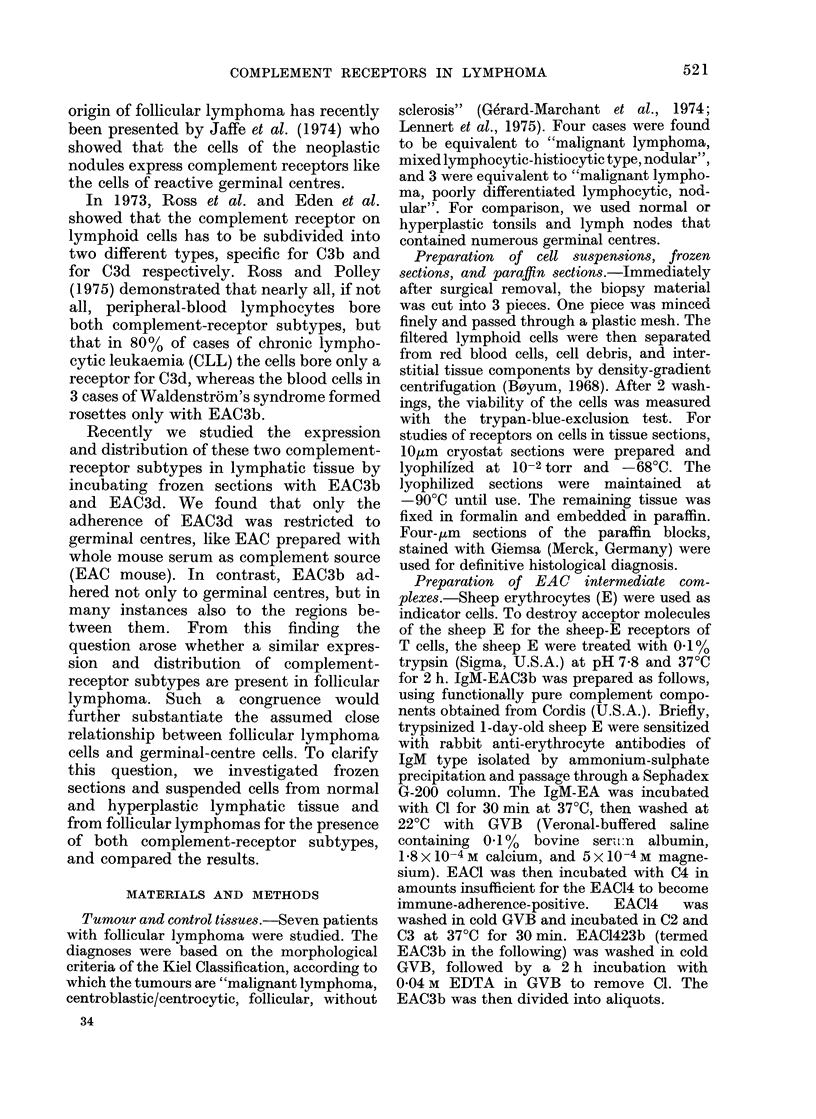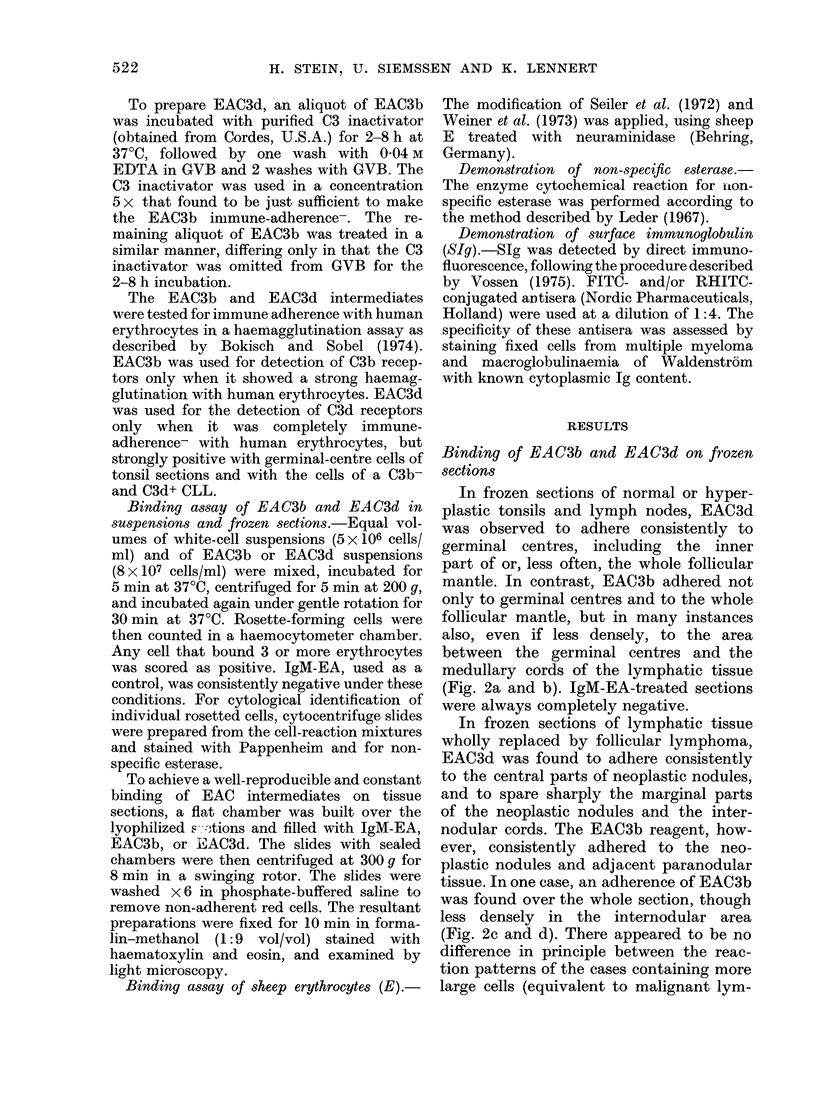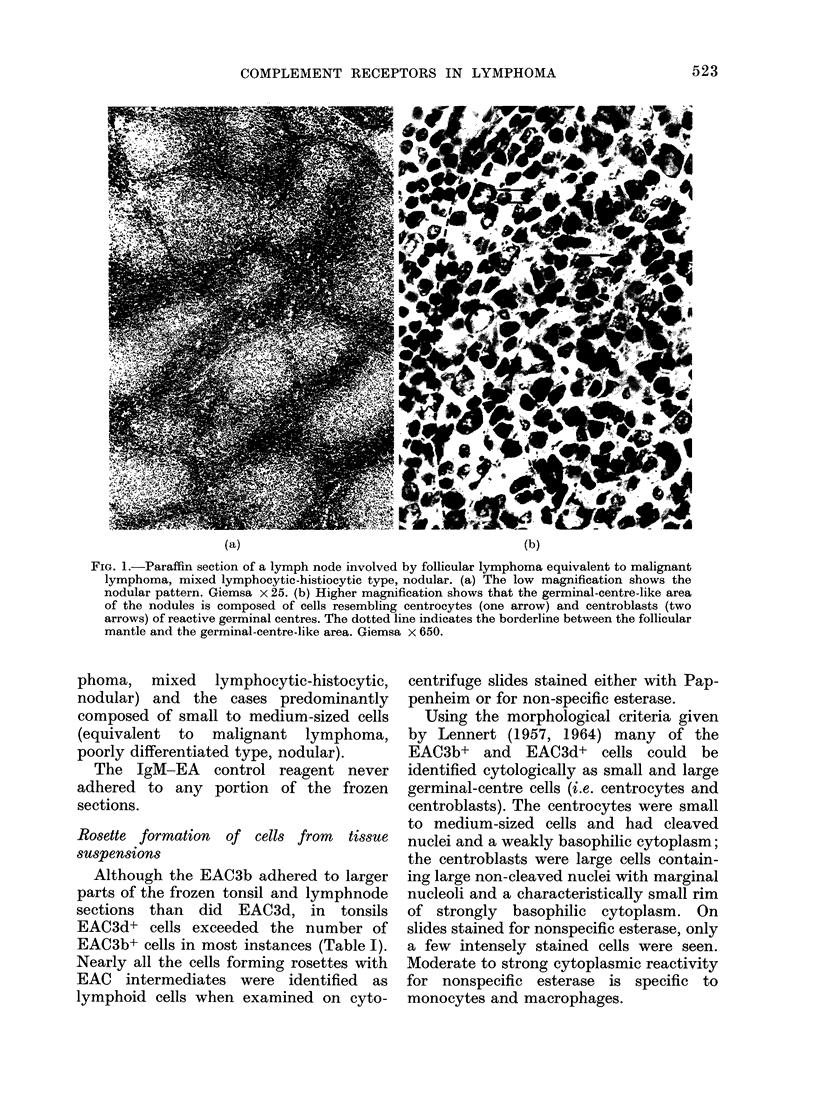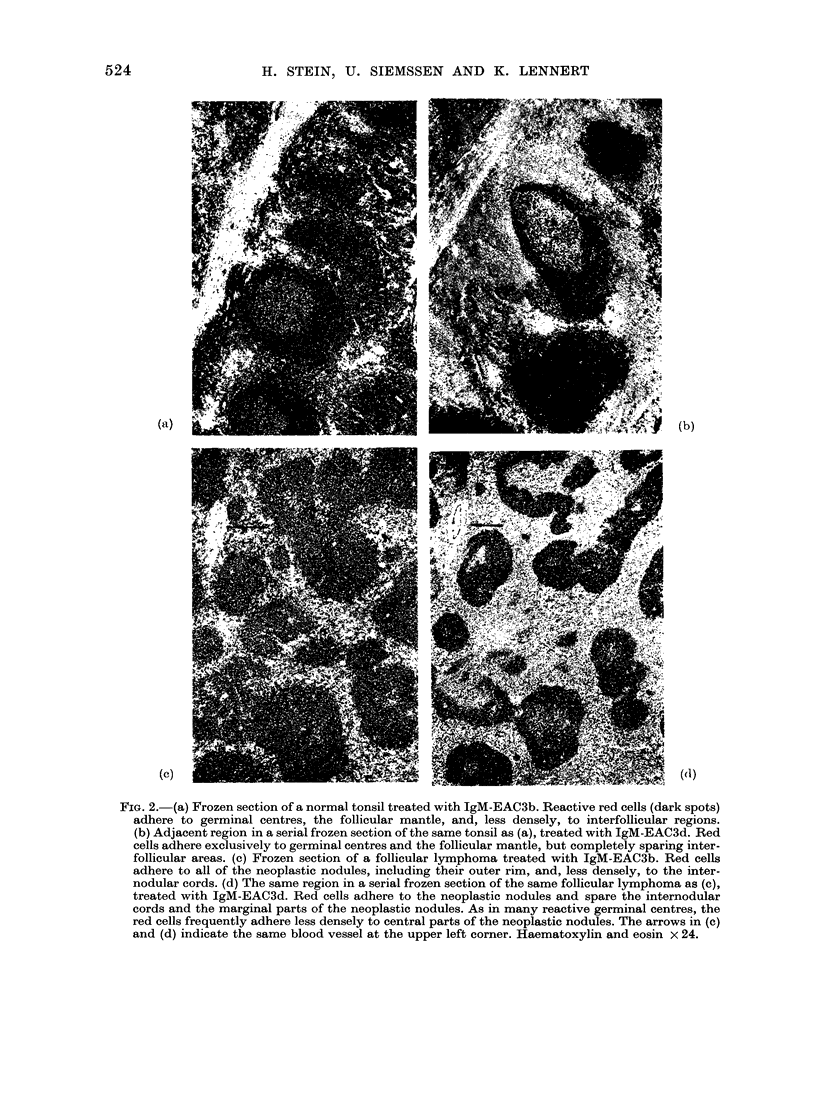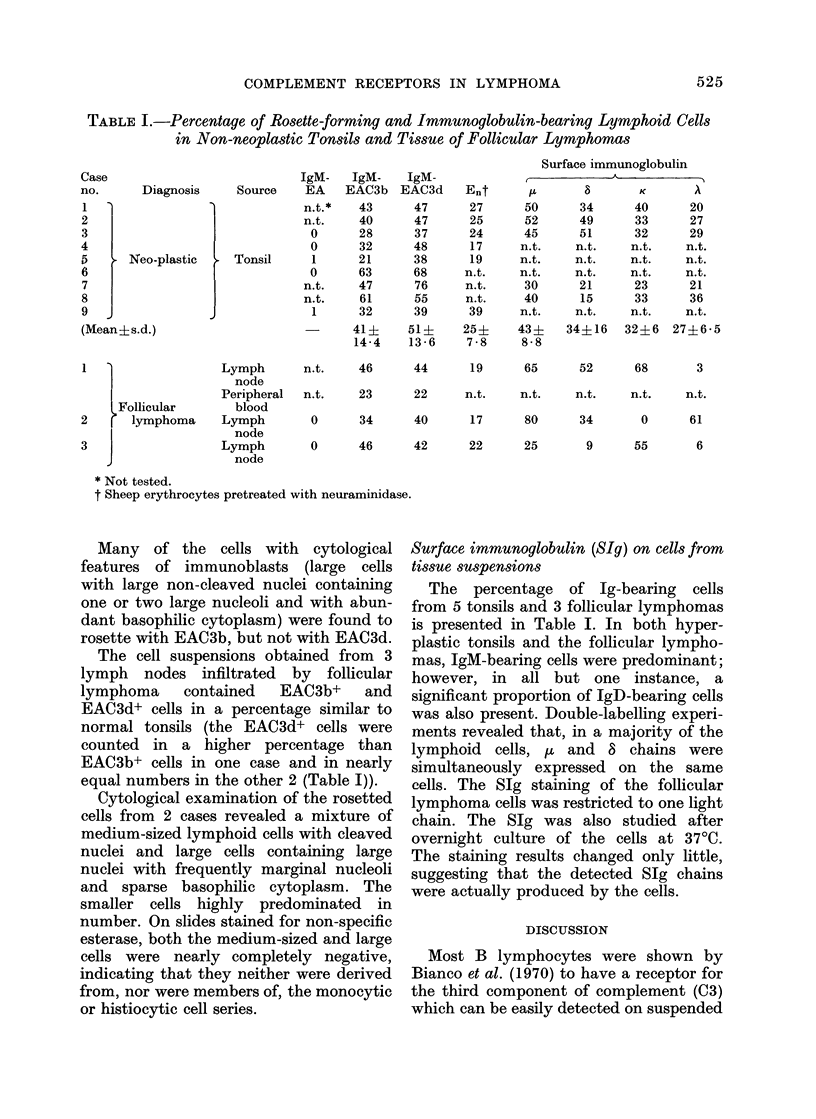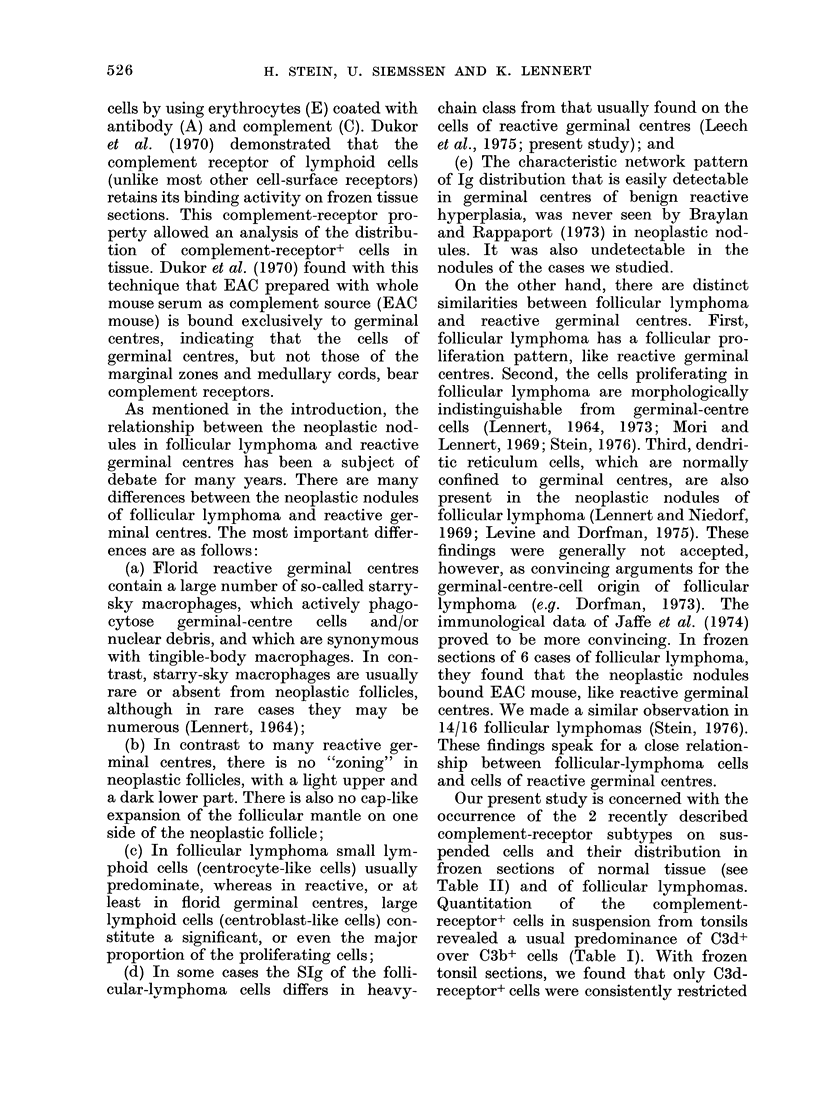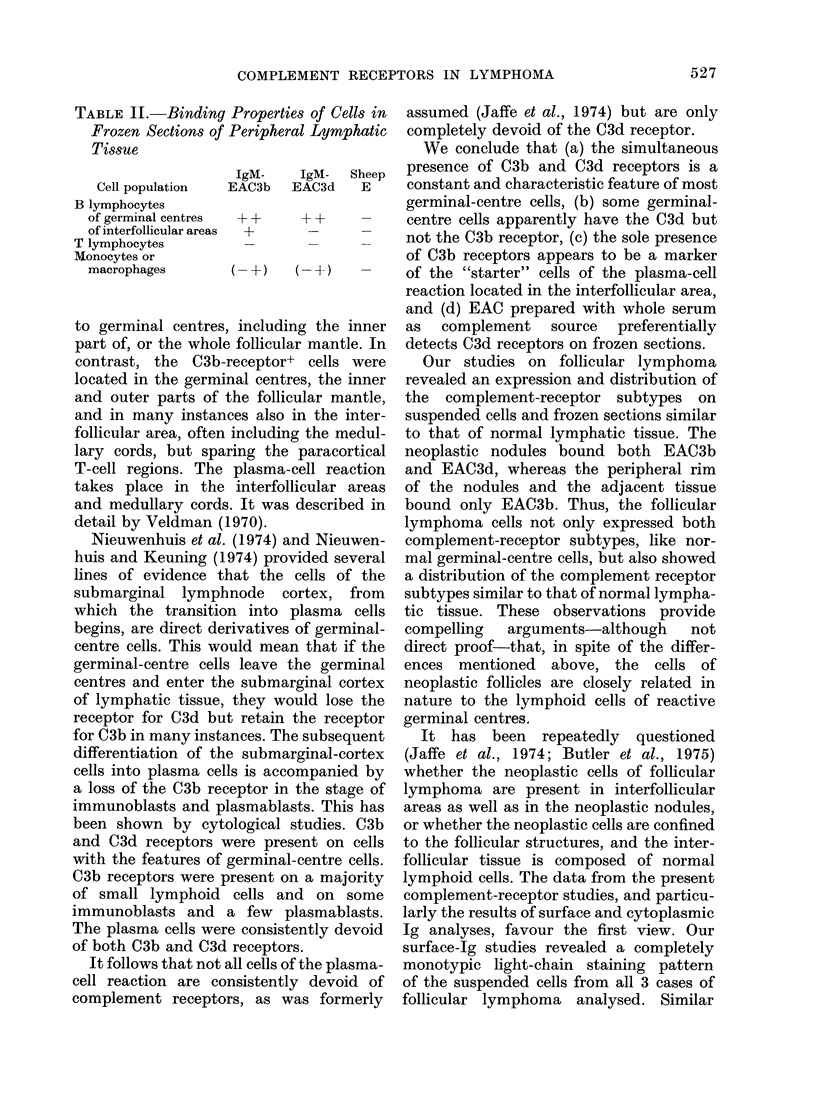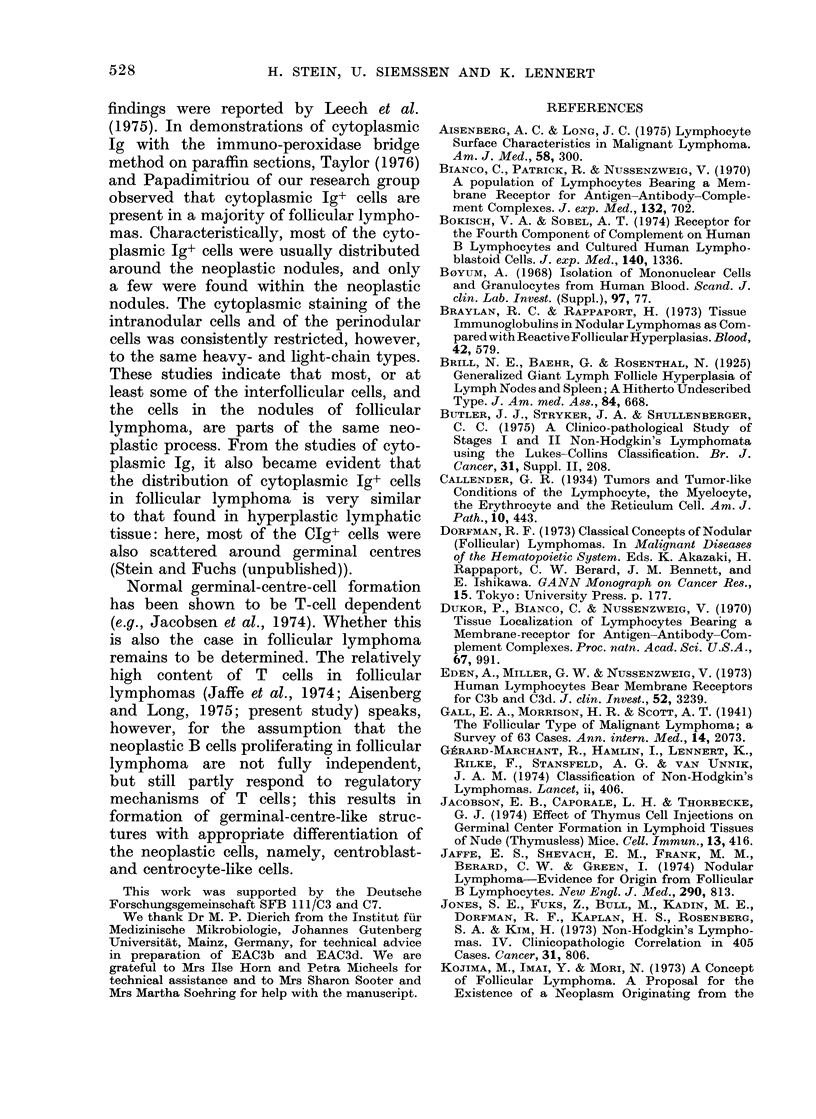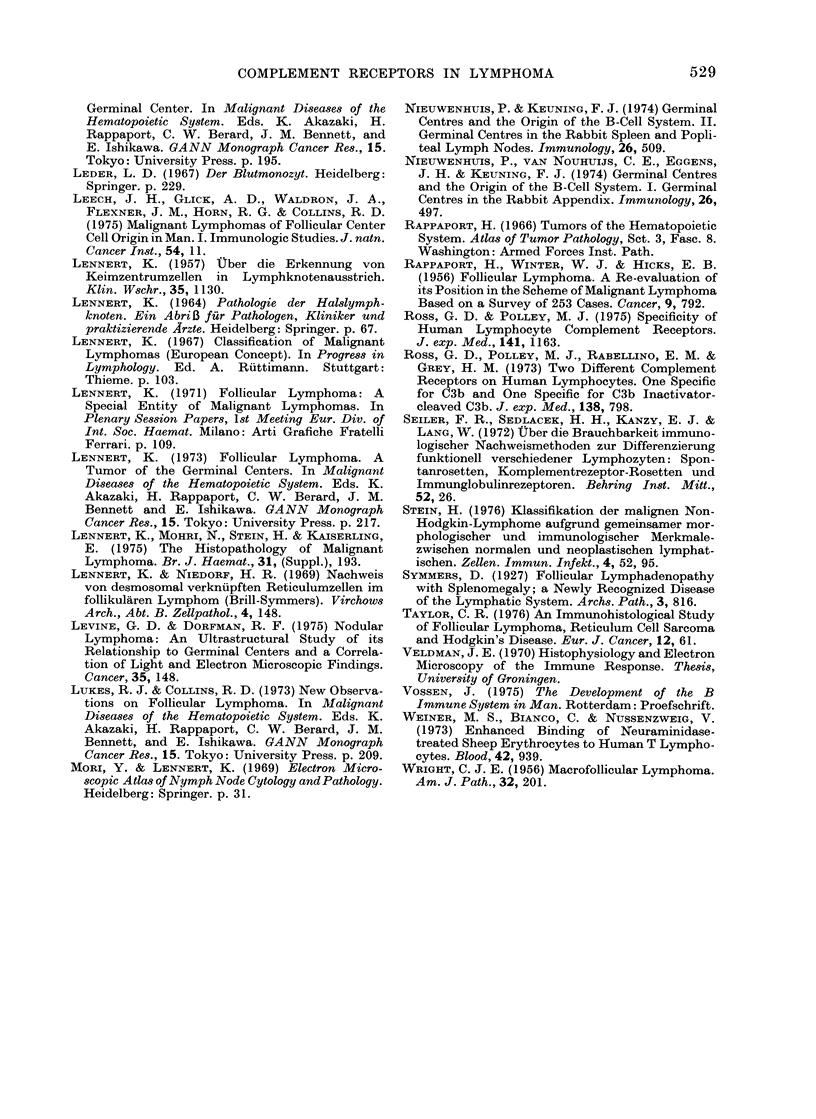# Complement Receptor Subtypes C3b and C3d in Lymphatic Tissue and Follicular Lymphoma

**DOI:** 10.1038/bjc.1978.80

**Published:** 1978-04

**Authors:** H. Stein, U. Siemssen, K. Lennert

## Abstract

**Images:**


*Br. J. Cancer* (1978) **37**, 520

### COMPLEMENT RECEPTOR SUBTYPES C3b AND C3d IN LYMPHATIC TISSUE AND FOLLICULAR LYMPHOMA

H. STEIN, U. SIEMSSEN AND K. LENNERT

*From the Department of General Pathology and Pathologic Anatomy, University of Kiel, Hospitalstr. 42, D-2300 Kiel, Federal Republic of Germany*

Received 27 October 1977 Accepted 20 December 1977

**Summary.—To substantiate the origin of follicular (nodular) lymphoma cells from germinal-centre cells, the lymphoma cells from 7 patients with follicular lymphoma and from 9 tonsils and 2 lymph nodes were studied for the presence and distribution of complement-receptor subtypes (*i.e.*, the receptors for C3b and C3d). It was found that erythrocytes coated with antibodies and C3d (EAC3d) adhered exclusively to germinal centres, whereas erythrocytes coated with antibodies and C3b (EAC3b) adhered to germinal centres and in many instances to the regions between them. These findings indicate that germinal-centre cells bear both complement-receptor subtypes and that the B cells of the interfollicular area, which belong at least in part to the precursors of plasma cells, bear only a receptor for C3b. In frozen sections of follicular lymphomas, a similar distribution of complement-receptor subtypes was observed; EAC3d was bound exclusively to the neoplastic nodules, and EAC3b adhered to the neoplastic nodules and adjacent paranodular tissue. Receptor studies on suspended cells of both normal tonsils and follicular lymphomas revealed a slight predominance of EAC3d$^+$ cells or equal numbers of EAC3b$^+$ and EAC3d$^+$ cells. The complete congruence in the expression and |distribution of complement-receptor subtypes between tissues from follicular lymphomas and those from normal and hyperplastic tonsils or lymph nodes suggests that follicular lymphoma represents the neoplastic counterpart of the reactive germinal centre.**

FOLLICULAR (nodular) lymphoma is the most common subgroup of malignant non-Hodgkin's lymphoma. Until recently, the cellular origin of the follicular lymphoma cells had been controversial. Brill *et al.* (1925) and Symmers (1927) regarded the nodules as hyperplastic follicles. Later studies established their neoplastic nature (Callender, 1934; Gall *et al.*, 1941; Wright, 1956). Rappaport *et al.* (1956) maintained that there was no conclusive evidence for the contention that the so-called follicles of follicular lymphoma arose from, or were related to, reactive germinal centres. Because of the morphological variations observed within this lymphoma group, these authors considered the nodular growth pattern to be an architectural variant that could be seen in any of the histological types of non-Hodgkin's lymphoma. This concept has been widely accepted and, accordingly, the term "nodular lymphoma" was preferred (Rappaport, 1966; Dorfman, 1973; Jones *et al.*, 1973).

Other authors (Lennert, 1964, 1967, 1971, 1973; Mori and Lennert, 1969; Kojima *et al.*, 1973; Lukes and Collins, 1973) however, pointed out the similarity between the cells of follicular lymphomas and germinal centres, and regarded follicular lymphoma as a distinct entity originating from germinal-centre cells. This view was substantiated by the detection of dendritic reticulum cells in follicular lymphoma (Lennert and Niedorf, 1969; Levine and Dorfman, 1975). Strong evidence for the germinal-centre cell origin of follicular lymphoma has recently been presented by Jaffe *et al.* (1974) who showed that the cells of the neoplastic nodules express complement receptors like the cells of reactive germinal centres.

In 1973, Ross *et al.* and Eden *et al.* showed that the complement receptor on lymphoid cells has to be subdivided into two different types, specific for C3b and for C3d respectively. Ross and Polley (1975) demonstrated that nearly all, if not all, peripheral-blood lymphocytes bore both complement-receptor subtypes, but that in 80% of cases of chronic lymphocytic leukaemia (CLL) the cells bore only a receptor for C3d, whereas the blood cells in 3 cases of Waldenström's syndrome formed rosettes only with EAC3b.

Recently we studied the expression and distribution of these two complement-receptor subtypes in lymphatic tissue by incubating frozen sections with EAC3b and EAC3d. We found that only the adherence of EAC3d was restricted to germinal centres, like EAC prepared with whole mouse serum as complement source (EAC mouse). In contrast, EAC3b adhered not only to germinal centres, but in many instances also to the regions between them. From this finding the question arose whether a similar expression and distribution of complement-receptor subtypes are present in follicular lymphoma. Such a congruence would further substantiate the assumed close relationship between follicular lymphoma cells and germinal-centre cells. To clarify this question, we investigated frozen sections and suspended cells from normal and hyperplastic lymphatic tissue and from follicular lymphomas for the presence of both complement-receptor subtypes, and compared the results.

### MATERIALS AND METHODS

*Tumour and control tissues.*—Seven patients with follicular lymphoma were studied. The diagnoses were based on the morphological criteria of the Kiel Classification, according to which the tumours are "malignant lymphoma, centroblastic/centrocytic, follicular, without sclerosis" (Gérard-Marchant *et al.*, 1974; Lennert *et al.*, 1975). Four cases were found to be equivalent to "malignant lymphoma, mixed lymphocytic-histiocytic type, nodular", and 3 were equivalent to "malignant lymphoma, poorly differentiated lymphocytic, nodular". For comparison, we used normal or hyperplastic tonsils and lymph nodes that contained numerous germinal centres.

*Preparation of cell suspensions, frozen sections, and paraffin sections.*—Immediately after surgical removal, the biopsy material was cut into 3 pieces. One piece was minced finely and passed through a plastic mesh. The filtered lymphoid cells were then separated from red blood cells, cell debris, and interstitial tissue components by density-gradient centrifugation (Bøyum, 1968). After 2 washings, the viability of the cells was measured with the trypan-blue-exclusion test. For studies of receptors on cells in tissue sections, 10$\mu$m cryostat sections were prepared and lyophilized at $10^{-2}$ torr and $-68$°C. The lyophilized sections were maintained at $-90$°C until use. The remaining tissue was fixed in formalin and embedded in paraffin. Four-$\mu$m sections of the paraffin blocks, stained with Giemsa (Merck, Germany) were used for definitive histological diagnosis.

*Preparation of EAC intermediate complexes.*—Sheep erythrocytes (E) were used as indicator cells. To destroy acceptor molecules of the sheep E for the sheep-E receptors of T cells, the sheep E were treated with 0·1% trypsin (Sigma, U.S.A.) at pH 7·8 and 37°C for 2 h. IgM-EAC3b was prepared as follows, using functionally pure complement components obtained from Cordis (U.S.A.). Briefly, trypsinized 1-day-old sheep E were sensitized with rabbit anti-erythrocyte antibodies of IgM type isolated by ammonium-sulphate precipitation and passage through a Sephadex G-200 column. The IgM-EA was incubated with Cl for 30 min at 37°C, then washed at 22°C with GVB (Veronal-buffered saline containing 0·1% bovine serum albumin, $1{\cdot}8 \times 10^{-4}$ M calcium, and $5 \times 10^{-4}$ M magnesium). EACl was then incubated with C4 in amounts insufficient for the EACl4 to become immune-adherence-positive. EACl4 was washed in cold GVB and incubated in C2 and C3 at 37°C for 30 min. EACl423b (termed EAC3b in the following) was washed in cold GVB, followed by a 2 h incubation with 0·04 M EDTA in GVB to remove Cl. The EAC3b was then divided into aliquots.

To prepare EAC3d, an aliquot of EAC3b was incubated with purified C3 inactivator (obtained from Cordes, U.S.A.) for 2–8 h at 37°C, followed by one wash with 0·04 M EDTA in GVB and 2 washes with GVB. The C3 inactivator was used in a concentration 5× that found to be just sufficient to make the EAC3b immune-adherence⁻. The remaining aliquot of EAC3b was treated in a similar manner, differing only in that the C3 inactivator was omitted from GVB for the 2–8 h incubation.

The EAC3b and EAC3d intermediates were tested for immune adherence with human erythrocytes in a haemagglutination assay as described by Bokisch and Sobel (1974). EAC3b was used for detection of C3b receptors only when it showed a strong haemagglutination with human erythrocytes. EAC3d was used for the detection of C3d receptors only when it was completely immune-adherence⁻ with human erythrocytes, but strongly positive with germinal-centre cells of tonsil sections and with the cells of a C3b⁻ and C3d⁺ CLL.

*Binding assay of EAC3b and EAC3d in suspensions and frozen sections.*—Equal volumes of white-cell suspensions ($5\times10^6$ cells/ml) and of EAC3b or EAC3d suspensions ($8\times10^7$ cells/ml) were mixed, incubated for 5 min at 37°C, centrifuged for 5 min at 200 *g*, and incubated again under gentle rotation for 30 min at 37°C. Rosette-forming cells were then counted in a haemocytometer chamber. Any cell that bound 3 or more erythrocytes was scored as positive. IgM-EA, used as a control, was consistently negative under these conditions. For cytological identification of individual rosetted cells, cytocentrifuge slides were prepared from the cell-reaction mixtures and stained with Pappenheim and for non-specific esterase.

To achieve a well-reproducible and constant binding of EAC intermediates on tissue sections, a flat chamber was built over the lyophilized sections and filled with IgM-EA, EAC3b, or EAC3d. The slides with sealed chambers were then centrifuged at 300 *g* for 8 min in a swinging rotor. The slides were washed ×6 in phosphate-buffered saline to remove non-adherent red cells. The resultant preparations were fixed for 10 min in formalin–methanol (1:9 vol/vol) stained with haematoxylin and eosin, and examined by light microscopy.

*Binding assay of sheep erythrocytes (E).*—The modification of Seiler *et al.* (1972) and Weiner *et al.* (1973) was applied, using sheep E treated with neuraminidase (Behring, Germany).

*Demonstration of non-specific esterase.*—The enzyme cytochemical reaction for non-specific esterase was performed according to the method described by Leder (1967).

*Demonstration of surface immunoglobulin (SIg).*—SIg was detected by direct immunofluorescence, following the procedure described by Vossen (1975). FITC- and/or RHITC-conjugated antisera (Nordic Pharmaceuticals, Holland) were used at a dilution of 1:4. The specificity of these antisera was assessed by staining fixed cells from multiple myeloma and macroglobulinaemia of Waldenström with known cytoplasmic Ig content.

### RESULTS

### *Binding of EAC3b and EAC3d on frozen sections*

In frozen sections of normal or hyperplastic tonsils and lymph nodes, EAC3d was observed to adhere consistently to germinal centres, including the inner part of or, less often, the whole follicular mantle. In contrast, EAC3b adhered not only to germinal centres and to the whole follicular mantle, but in many instances also, even if less densely, to the area between the germinal centres and the medullary cords of the lymphatic tissue (Fig. 2a and b). IgM-EA-treated sections were always completely negative.

In frozen sections of lymphatic tissue wholly replaced by follicular lymphoma, EAC3d was found to adhere consistently to the central parts of neoplastic nodules, and to spare sharply the marginal parts of the neoplastic nodules and the internodular cords. The EAC3b reagent, however, consistently adhered to the neoplastic nodules and adjacent paranodular tissue. In one case, an adherence of EAC3b was found over the whole section, though less densely in the internodular area (Fig. 2c and d). There appeared to be no difference in principle between the reaction patterns of the cases containing more large cells (equivalent to malignant lym-

FIG. 1.—Paraffin section of a lymph node involved by follicular lymphoma equivalent to malignant lymphoma, mixed lymphocytic-histiocytic type, nodular. (a) The low magnification shows the nodular pattern. Giemsa ×25. (b) Higher magnification shows that the germinal-centre-like area of the nodules is composed of cells resembling centrocytes (one arrow) and centroblasts (two arrows) of reactive germinal centres. The dotted line indicates the borderline between the follicular mantle and the germinal-centre-like area. Giemsa ×650.

phoma, mixed lymphocytic-histocytic, nodular) and the cases predominantly composed of small to medium-sized cells (equivalent to malignant lymphoma, poorly differentiated type, nodular).

The IgM–EA control reagent never adhered to any portion of the frozen sections.

*Rosette formation of cells from tissue suspensions*

Although the EAC3b adhered to larger parts of the frozen tonsil and lymphnode sections than did EAC3d, in tonsils EAC3d$^+$ cells exceeded the number of EAC3b$^+$ cells in most instances (Table I). Nearly all the cells forming rosettes with EAC intermediates were identified as lymphoid cells when examined on cyto-centrifuge slides stained either with Pappenheim or for non-specific esterase.

Using the morphological criteria given by Lennert (1957, 1964) many of the EAC3b$^+$ and EAC3d$^+$ cells could be identified cytologically as small and large germinal-centre cells (*i.e.* centrocytes and centroblasts). The centrocytes were small to medium-sized cells and had cleaved nuclei and a weakly basophilic cytoplasm; the centroblasts were large cells containing large non-cleaved nuclei with marginal nucleoli and a characteristically small rim of strongly basophilic cytoplasm. On slides stained for nonspecific esterase, only a few intensely stained cells were seen. Moderate to strong cytoplasmic reactivity for nonspecific esterase is specific to monocytes and macrophages.

FIG. 2.—(a) Frozen section of a normal tonsil treated with IgM-EAC3b. Reactive red cells (dark spots) adhere to germinal centres, the follicular mantle, and, less densely, to interfollicular regions. (b) Adjacent region in a serial frozen section of the same tonsil as (a), treated with IgM-EAC3d. Red cells adhere exclusively to germinal centres and the follicular mantle, but completely sparing interfollicular areas. (c) Frozen section of a follicular lymphoma treated with IgM-EAC3b. Red cells adhere to all of the neoplastic nodules, including their outer rim, and, less densely, to the internodular cords. (d) The same region in a serial frozen section of the same follicular lymphoma as (c), treated with IgM-EAC3d. Red cells adhere to the neoplastic nodules and spare the internodular cords and the marginal parts of the neoplastic nodules. As in many reactive germinal centres, the red cells frequently adhere less densely to central parts of the neoplastic nodules. The arrows in (c) and (d) indicate the same blood vessel at the upper left corner. Haematoxylin and eosin $\times 24$.

TABLE I.—*Percentage of Rosette-forming and Immunoglobulin-bearing Lymphoid Cells in Non-neoplastic Tonsils and Tissue of Follicular Lymphomas*

| Case no. | Diagnosis | Source | IgM-EA | IgM-EAC3b | IgM-EAC3d | $E_n$† | Surface immunoglobulin $\mu$ | $\delta$ | $\kappa$ | $\lambda$ |
|---|---|---|---|---|---|---|---|---|---|---|
| 1 | Neo-plastic | Tonsil | n.t.* | 43 | 47 | 27 | 50 | 34 | 40 | 20 |
| 2 | | | n.t. | 40 | 47 | 25 | 52 | 49 | 33 | 27 |
| 3 | | | 0 | 28 | 37 | 24 | 45 | 51 | 32 | 29 |
| 4 | | | 0 | 32 | 48 | 17 | n.t. | n.t. | n.t. | n.t. |
| 5 | | | 1 | 21 | 38 | 19 | n.t. | n.t. | n.t. | n.t. |
| 6 | | | 0 | 63 | 68 | n.t. | n.t. | n.t. | n.t. | n.t. |
| 7 | | | n.t. | 47 | 76 | n.t. | 30 | 21 | 23 | 21 |
| 8 | | | n.t. | 61 | 55 | n.t. | 40 | 15 | 33 | 36 |
| 9 | | | 1 | 32 | 39 | 39 | n.t. | n.t. | n.t. | n.t. |
| (Mean ± s.d.) | | | — | 41 ± 14·4 | 51 ± 13·6 | 25 ± 7·8 | 43 ± 8·8 | 34 ± 16 | 32 ± 6 | 27 ± 6·5 |
| 1 | Follicular lymphoma | Lymph node | n.t. | 46 | 44 | 19 | 65 | 52 | 68 | 3 |
| | | Peripheral blood | n.t. | 23 | 22 | n.t. | n.t. | n.t. | n.t. | n.t. |
| 2 | | Lymph node | 0 | 34 | 40 | 17 | 80 | 34 | 0 | 61 |
| 3 | | Lymph node | 0 | 46 | 42 | 22 | 25 | 9 | 55 | 6 |

* Not tested.
† Sheep erythrocytes pretreated with neuraminidase.

Many of the cells with cytological features of immunoblasts (large cells with large non-cleaved nuclei containing one or two large nucleoli and with abundant basophilic cytoplasm) were found to rosette with EAC3b, but not with EAC3d.

The cell suspensions obtained from 3 lymph nodes infiltrated by follicular lymphoma contained $EAC3b^+$ and $EAC3d^+$ cells in a percentage similar to normal tonsils (the $EAC3d^+$ cells were counted in a higher percentage than $EAC3b^+$ cells in one case and in nearly equal numbers in the other 2 (Table I)).

Cytological examination of the rosetted cells from 2 cases revealed a mixture of medium-sized lymphoid cells with cleaved nuclei and large cells containing large nuclei with frequently marginal nucleoli and sparse basophilic cytoplasm. The smaller cells highly predominated in number. On slides stained for non-specific esterase, both the medium-sized and large cells were nearly completely negative, indicating that they neither were derived from, nor were members of, the monocytic or histiocytic cell series.

*Surface immunoglobulin (SIg) on cells from tissue suspensions*

The percentage of Ig-bearing cells from 5 tonsils and 3 follicular lymphomas is presented in Table I. In both hyperplastic tonsils and the follicular lymphomas, IgM-bearing cells were predominant; however, in all but one instance, a significant proportion of IgD-bearing cells was also present. Double-labelling experiments revealed that, in a majority of the lymphoid cells, $\mu$ and $\delta$ chains were simultaneously expressed on the same cells. The SIg staining of the follicular lymphoma cells was restricted to one light chain. The SIg was also studied after overnight culture of the cells at 37°C. The staining results changed only little, suggesting that the detected SIg chains were actually produced by the cells.

### DISCUSSION

Most B lymphocytes were shown by Bianco *et al.* (1970) to have a receptor for the third component of complement (C3) which can be easily detected on suspended cells by using erythrocytes (E) coated with antibody (A) and complement (C). Dukor *et al.* (1970) demonstrated that the complement receptor of lymphoid cells (unlike most other cell-surface receptors) retains its binding activity on frozen tissue sections. This complement-receptor property allowed an analysis of the distribution of complement-receptor$^+$ cells in tissue. Dukor *et al.* (1970) found with this technique that EAC prepared with whole mouse serum as complement source (EAC mouse) is bound exclusively to germinal centres, indicating that the cells of germinal centres, but not those of the marginal zones and medullary cords, bear complement receptors.

As mentioned in the introduction, the relationship between the neoplastic nodules in follicular lymphoma and reactive germinal centres has been a subject of debate for many years. There are many differences between the neoplastic nodules of follicular lymphoma and reactive germinal centres. The most important differences are as follows:

(a) Florid reactive germinal centres contain a large number of so-called starry-sky macrophages, which actively phagocytose germinal-centre cells and/or nuclear debris, and which are synonymous with tingible-body macrophages. In contrast, starry-sky macrophages are usually rare or absent from neoplastic follicles, although in rare cases they may be numerous (Lennert, 1964);

(b) In contrast to many reactive germinal centres, there is no "zoning" in neoplastic follicles, with a light upper and a dark lower part. There is also no cap-like expansion of the follicular mantle on one side of the neoplastic follicle;

(c) In follicular lymphoma small lymphoid cells (centrocyte-like cells) usually predominate, whereas in reactive, or at least in florid germinal centres, large lymphoid cells (centroblast-like cells) constitute a significant, or even the major proportion of the proliferating cells;

(d) In some cases the SIg of the follicular-lymphoma cells differs in heavy-chain class from that usually found on the cells of reactive germinal centres (Leech *et al.*, 1975; present study); and

(e) The characteristic network pattern of Ig distribution that is easily detectable in germinal centres of benign reactive hyperplasia, was never seen by Braylan and Rappaport (1973) in neoplastic nodules. It was also undetectable in the nodules of the cases we studied.

On the other hand, there are distinct similarities between follicular lymphoma and reactive germinal centres. First, follicular lymphoma has a follicular proliferation pattern, like reactive germinal centres. Second, the cells proliferating in follicular lymphoma are morphologically indistinguishable from germinal-centre cells (Lennert, 1964, 1973; Mori and Lennert, 1969; Stein, 1976). Third, dendritic reticulum cells, which are normally confined to germinal centres, are also present in the neoplastic nodules of follicular lymphoma (Lennert and Niedorf, 1969; Levine and Dorfman, 1975). These findings were generally not accepted, however, as convincing arguments for the germinal-centre-cell origin of follicular lymphoma (*e.g.* Dorfman, 1973). The immunological data of Jaffe *et al.* (1974) proved to be more convincing. In frozen sections of 6 cases of follicular lymphoma, they found that the neoplastic nodules bound EAC mouse, like reactive germinal centres. We made a similar observation in 14/16 follicular lymphomas (Stein, 1976). These findings speak for a close relationship between follicular-lymphoma cells and cells of reactive germinal centres.

Our present study is concerned with the occurrence of the 2 recently described complement-receptor subtypes on suspended cells and their distribution in frozen sections of normal tissue (see Table II) and of follicular lymphomas. Quantitation of the complement-receptor$^+$ cells in suspension from tonsils revealed a usual predominance of C3d$^+$ over C3b$^+$ cells (Table I). With frozen tonsil sections, we found that only C3d-receptor$^+$ cells were consistently restricted

TABLE II.—*Binding Properties of Cells in Frozen Sections of Peripheral Lymphatic Tissue*

| Cell population | IgM-EAC3b | IgM-EAC3d | Sheep E |
|---|---|---|---|
| B lymphocytes | | | |
| of germinal centres | ++ | ++ | − |
| of interfollicular areas | + | − | − |
| T lymphocytes | − | − | − |
| Monocytes or macrophages | (−+) | (−+) | − |

to germinal centres, including the inner part of, or the whole follicular mantle. In contrast, the C3b-receptor$^+$ cells were located in the germinal centres, the inner and outer parts of the follicular mantle, and in many instances also in the interfollicular area, often including the medullary cords, but sparing the paracortical T-cell regions. The plasma-cell reaction takes place in the interfollicular areas and medullary cords. It was described in detail by Veldman (1970).

Nieuwenhuis *et al.* (1974) and Nieuwenhuis and Keuning (1974) provided several lines of evidence that the cells of the submarginal lymphnode cortex, from which the transition into plasma cells begins, are direct derivatives of germinal-centre cells. This would mean that if the germinal-centre cells leave the germinal centres and enter the submarginal cortex of lymphatic tissue, they would lose the receptor for C3d but retain the receptor for C3b in many instances. The subsequent differentiation of the submarginal-cortex cells into plasma cells is accompanied by a loss of the C3b receptor in the stage of immunoblasts and plasmablasts. This has been shown by cytological studies. C3b and C3d receptors were present on cells with the features of germinal-centre cells. C3b receptors were present on a majority of small lymphoid cells and on some immunoblasts and a few plasmablasts. The plasma cells were consistently devoid of both C3b and C3d receptors.

It follows that not all cells of the plasma-cell reaction are consistently devoid of complement receptors, as was formerly assumed (Jaffe *et al.*, 1974) but are only completely devoid of the C3d receptor.

We conclude that (a) the simultaneous presence of C3b and C3d receptors is a constant and characteristic feature of most germinal-centre cells, (b) some germinal-centre cells apparently have the C3d but not the C3b receptor, (c) the sole presence of C3b receptors appears to be a marker of the "starter" cells of the plasma-cell reaction located in the interfollicular area, and (d) EAC prepared with whole serum as complement source preferentially detects C3d receptors on frozen sections.

Our studies on follicular lymphoma revealed an expression and distribution of the complement-receptor subtypes on suspended cells and frozen sections similar to that of normal lymphatic tissue. The neoplastic nodules bound both EAC3b and EAC3d, whereas the peripheral rim of the nodules and the adjacent tissue bound only EAC3b. Thus, the follicular lymphoma cells not only expressed both complement-receptor subtypes, like normal germinal-centre cells, but also showed a distribution of the complement receptor subtypes similar to that of normal lymphatic tissue. These observations provide compelling arguments—although not direct proof—that, in spite of the differences mentioned above, the cells of neoplastic follicles are closely related in nature to the lymphoid cells of reactive germinal centres.

It has been repeatedly questioned (Jaffe *et al.*, 1974; Butler *et al.*, 1975) whether the neoplastic cells of follicular lymphoma are present in interfollicular areas as well as in the neoplastic nodules, or whether the neoplastic cells are confined to the follicular structures, and the interfollicular tissue is composed of normal lymphoid cells. The data from the present complement-receptor studies, and particularly the results of surface and cytoplasmic Ig analyses, favour the first view. Our surface-Ig studies revealed a completely monotypic light-chain staining pattern of the suspended cells from all 3 cases of follicular lymphoma analysed. Similar findings were reported by Leech *et al.* (1975). In demonstrations of cytoplasmic Ig with the immuno-peroxidase bridge method on paraffin sections, Taylor (1976) and Papadimitriou of our research group observed that cytoplasmic $Ig^+$ cells are present in a majority of follicular lymphomas. Characteristically, most of the cytoplasmic $Ig^+$ cells were usually distributed around the neoplastic nodules, and only a few were found within the neoplastic nodules. The cytoplasmic staining of the intranodular cells and of the perinodular cells was consistently restricted, however, to the same heavy- and light-chain types. These studies indicate that most, or at least some of the interfollicular cells, and the cells in the nodules of follicular lymphoma, are parts of the same neoplastic process. From the studies of cytoplasmic Ig, it also became evident that the distribution of cytoplasmic $Ig^+$ cells in follicular lymphoma is very similar to that found in hyperplastic lymphatic tissue: here, most of the $CIg^+$ cells were also scattered around germinal centres (Stein and Fuchs (unpublished)).

Normal germinal-centre-cell formation has been shown to be T-cell dependent (*e.g.*, Jacobsen *et al.*, 1974). Whether this is also the case in follicular lymphoma remains to be determined. The relatively high content of T cells in follicular lymphomas (Jaffe *et al.*, 1974; Aisenberg and Long, 1975; present study) speaks, however, for the assumption that the neoplastic B cells proliferating in follicular lymphoma are not fully independent, but still partly respond to regulatory mechanisms of T cells; this results in formation of germinal-centre-like structures with appropriate differentiation of the neoplastic cells, namely, centroblast- and centrocyte-like cells.

This work was supported by the Deutsche Forschungsgemeinschaft SFB 111/C3 and C7.

We thank Dr M. P. Dierich from the Institut für Medizinische Mikrobiologie, Johannes Gutenberg Universität, Mainz, Germany, for technical advice in preparation of EAC3b and EAC3d. We are grateful to Mrs Ilse Horn and Petra Micheels for technical assistance and to Mrs Sharon Sooter and Mrs Martha Soehring for help with the manuscript.

### REFERENCES

Aisenberg, A. C. & Long, J. C. (1975) Lymphocyte Surface Characteristics in Malignant Lymphoma. *Am. J. Med.*, **58**, 300.

Bianco, C., Patrick, R. & Nussenzweig, V. (1970) A population of Lymphocytes Bearing a Membrane Receptor for Antigen–Antibody–Complement Complexes. *J. exp. Med.*, **132**, 702.

Bokisch, V. A. & Sobel, A. T. (1974) Receptor for the Fourth Component of Complement on Human B Lymphocytes and Cultured Human Lymphoblastoid Cells. *J. exp. Med.*, **140**, 1336.

Bøyum, A. (1968) Isolation of Mononuclear Cells and Granulocytes from Human Blood. *Scand. J. clin. Lab. Invest.* (Suppl.), **97**, 77.

Braylan, R. C. & Rappaport, H. (1973) Tissue Immunoglobulins in Nodular Lymphomas as Compared with Reactive Follicular Hyperplasias. *Blood*, **42**, 579.

Brill, N. E., Baehr, G. & Rosenthal, N. (1925) Generalized Giant Lymph Follicle Hyperplasia of Lymph Nodes and Spleen; A Hitherto Undescribed Type. *J. Am. med. Ass.*, **84**, 668.

Butler, J. J., Stryker, J. A. & Shullenberger, C. C. (1975) A Clinico-pathological Study of Stages I and II Non-Hodgkin's Lymphomata using the Lukes–Collins Classification. *Br. J. Cancer*, **31**, Suppl. II, 208.

Callender, G. R. (1934) Tumors and Tumor-like Conditions of the Lymphocyte, the Myelocyte, the Erythrocyte and the Reticulum Cell. *Am. J. Path.*, **10**, 443.

Dorfman, R. F. (1973) Classical Concepts of Nodular (Follicular) Lymphomas. In *Malignant Diseases of the Hematopoietic System*. Eds. K. Akazaki, H. Rappaport, C. W. Berard, J. M. Bennett, and E. Ishikawa. *GANN Monograph on Cancer Res.*, **15**. Tokyo: University Press. p. 177.

Dukor, P., Bianco, C. & Nussenzweig, V. (1970) Tissue Localization of Lymphocytes Bearing a Membrane-receptor for Antigen–Antibody–Complement Complexes. *Proc. natn. Acad. Sci. U.S.A.*, **67**, 991.

Eden, A., Miller, G. W. & Nussenzweig, V. (1973) Human Lymphocytes Bear Membrane Receptors for C3b and C3d. *J. clin. Invest.*, **52**, 3239.

Gall, E. A., Morrison, H. R. & Scott, A. T. (1941) The Follicular Type of Malignant Lymphoma; a Survey of 63 Cases. *Ann. intern. Med.*, **14**, 2073.

Gérard-Marchant, R., Hamlin, I., Lennert, K., Rilke, F., Stansfeld, A. G. & van Unnik, J. A. M. (1974) Classification of Non-Hodgkin's Lymphomas. *Lancet*, ii, 406.

Jacobson, E. B., Caporale, L. H. & Thorbecke, G. J. (1974) Effect of Thymus Cell Injections on Germinal Center Formation in Lymphoid Tissues of Nude (Thymusless) Mice. *Cell. Immun.*, **13**, 416.

Jaffe, E. S., Shevach, E. M., Frank, M. M., Berard, C. W. & Green, I. (1974) Nodular Lymphoma—Evidence for Origin from Follicular B Lymphocytes. *New Engl. J. Med.*, **290**, 813.

Jones, S. E., Fuks, Z., Bull, M., Kadin, M. E., Dorfman, R. F., Kaplan, H. S., Rosenberg, S. A. & Kim, H. (1973) Non-Hodgkin's Lymphomas. IV. Clinicopathologic Correlation in 405 Cases. *Cancer*, **31**, 806.

Kojima, M., Imai, Y. & Mori, N. (1973) A Concept of Follicular Lymphoma. A Proposal for the Existence of a Neoplasm Originating from the

Germinal Center. In *Malignant Diseases of the Hematopoietic System.* Eds. K. Akazaki, H. Rappaport, C. W. Berard, J. M. Bennett, and E. Ishikawa. *GANN Monograph Cancer Res.*, **15**. Tokyo: University Press. p. 195.

LEDER, L. D. (1967) *Der Blutmonozyt.* Heidelberg: Springer. p. 229.

LEECH, J. H., GLICK, A. D., WALDRON, J. A., FLEXNER, J. M., HORN, R. G. & COLLINS, R. D. (1975) Malignant Lymphomas of Follicular Center Cell Origin in Man. I. Immunologic Studies. *J. natn. Cancer Inst.*, **54**, 11.

LENNERT, K. (1957) Über die Erkennung von Keimzentrumzellen in Lymphknotenausstrich. *Klin. Wschr.*, **35**, 1130.

LENNERT, K. (1964) *Pathologie der Halslymphknoten. Ein Abriß für Pathologen, Kliniker und praktizierende Ärzte.* Heidelberg: Springer. p. 67.

LENNERT, K. (1967) Classification of Malignant Lymphomas (European Concept). In *Progress in Lymphology.* Ed. A. Rüttimann. Stuttgart: Thieme. p. 103.

LENNERT, K. (1971) Follicular Lymphoma: A Special Entity of Malignant Lymphomas. In *Plenary Session Papers, 1st Meeting Eur. Div. of Int. Soc. Haemat.* Milano: Arti Grafiche Fratelli Ferrari. p. 109.

LENNERT, K. (1973) Follicular Lymphoma. A Tumor of the Germinal Centers. In *Malignant Diseases of the Hematopoietic System.* Eds. K. Akazaki, H. Rappaport, C. W. Berard, J. M. Bennett and E. Ishikawa. *GANN Monograph Cancer Res.*, **15**. Tokyo: University Press. p. 217.

LENNERT, K., MOHRI, N., STEIN, H. & KAISERLING, E. (1975) The Histopathology of Malignant Lymphoma. *Br. J. Haemat.*, **31**, (Suppl.), 193.

LENNERT, K. & NIEDORF, H. R. (1969) Nachweis von desmosomal verknüpften Reticulumzellen im follikulären Lymphom (Brill-Symmers). *Virchows Arch., Abt. B. Zellpathol.*, **4**, 148.

LEVINE, G. D. & DORFMAN, R. F. (1975) Nodular Lymphoma: An Ultrastructural Study of its Relationship to Germinal Centers and a Correlation of Light and Electron Microscopic Findings. *Cancer*, **35**, 148.

LUKES, R. J. & COLLINS, R. D. (1973) New Observations on Follicular Lymphoma. In *Malignant Diseases of the Hematopoietic System.* Eds. K. Akazaki, H. Rappaport, C. W. Berard, J. M. Bennett, and E. Ishikawa. *GANN Monograph Cancer Res.*, **15**. Tokyo: University Press. p. 209.

MORI, Y. & LENNERT, K. (1969) *Electron Microscopic Atlas of Nymph Node Cytology and Pathology.* Heidelberg: Springer. p. 31.

NIEUWENHUIS, P. & KEUNING, F. J. (1974) Germinal Centres and the Origin of the B-Cell System. II. Germinal Centres in the Rabbit Spleen and Popliteal Lymph Nodes. *Immunology*, **26**, 509.

NIEUWENHUIS, P., VAN NOUHUIJS, C. E., EGGENS, J. H. & KEUNING, F. J. (1974) Germinal Centres and the Origin of the B-Cell System. I. Germinal Centres in the Rabbit Appendix. *Immunology*, **26**, 497.

RAPPAPORT, H. (1966) Tumors of the Hematopoietic System. *Atlas of Tumor Pathology*, Sct. 3, Fasc. 8. Washington: Armed Forces Inst. Path.

RAPPAPORT, H., WINTER, W. J. & HICKS, E. B. (1956) Follicular Lymphoma. A Re-evaluation of its Position in the Scheme of Malignant Lymphoma Based on a Survey of 253 Cases. *Cancer*, **9**, 792.

ROSS, G. D. & POLLEY, M. J. (1975) Specificity of Human Lymphocyte Complement Receptors. *J. exp. Med.*, **141**, 1163.

ROSS, G. D., POLLEY, M. J., RABELLINO, E. M. & GREY, H. M. (1973) Two Different Complement Receptors on Human Lymphocytes. One Specific for C3b and One Specific for C3b Inactivator-cleaved C3b. *J. exp. Med.*, **138**, 798.

SEILER, F. R., SEDLACEK, H. H., KANZY, E. J. & LANG, W. (1972) Über die Brauchbarkeit immunologischer Nachweismethoden zur Differenzierung funktionell verschiedener Lymphozyten: Spontanrosetten, Komplementrezeptor-Rosetten und Immunglobulinrezeptoren. *Behring Inst. Mitt.*, **52**, 26.

STEIN, H. (1976) Klassifikation der malignen Non-Hodgkin-Lymphome aufgrund gemeinsamer morphologischer und immunologischer Merkmale-zwischen normalen und neoplastischen lymphatischen. *Zellen. Immun. Infekt.*, **4**, 52, 95.

SYMMERS, D. (1927) Follicular Lymphadenopathy with Splenomegaly; a Newly Recognized Disease of the Lymphatic System. *Archs. Path.*, **3**, 816.

TAYLOR, C. R. (1976) An Immunohistological Study of Follicular Lymphoma, Reticulum Cell Sarcoma and Hodgkin's Disease. *Eur. J. Cancer*, **12**, 61.

VELDMAN, J. E. (1970) Histophysiology and Electron Microscopy of the Immune Response. *Thesis, University of Groningen.*

VOSSEN, J. (1975) *The Development of the B Immune System in Man.* Rotterdam: Proefschrift.

WEINER, M. S., BIANCO, C. & NUSSENZWEIG, V. (1973) Enhanced Binding of Neuraminidase-treated Sheep Erythrocytes to Human T Lymphocytes. *Blood*, **42**, 939.

WRIGHT, C. J. E. (1956) Macrofollicular Lymphoma. *Am. J. Path.*, **32**, 201.